# High chlamydia infection and its associated factors among patients seeking clinic-based STI services in Southern China: A preliminary cross-sectional study

**DOI:** 10.3389/fpubh.2022.1005334

**Published:** 2022-11-23

**Authors:** Honglin Wang, Rongxing Weng, Chunlai Zhang, Jianbin Ye, Lizhang Wen, Jing Li, Yongyi Lu, Ning Ning, Fuchang Hong, Xiangsheng Chen, Yumao Cai

**Affiliations:** ^1^Department of STD and Leprosy Control and Prevention, Shenzhen Center for Chronic Disease Control, Shenzhen, China; ^2^Department of Communicable Diseases Control and Prevention, Shenzhen Center for Disease Control and Prevention, Shenzhen, China; ^3^Department of Health Studies, American University, Washington, DC, United States; ^4^Shantou University Medical College, Shantou University, Shantou, China; ^5^National Center for STD Control, China Center for Disease Control and Prevention, Nanjing, China

**Keywords:** chlamydia infection (CT), STI services, patients, Southern China, associated factors

## Abstract

**Objective:**

*Chlamydia trachomatis* (CT) infection is one of the most common sexually transmitted infections (STIs) worldwide. This study aimed to provide prevalence and associated factors data among patients seeking clinic-based STI services for estimating the disease burden of CT.

**Study design and method:**

A cross-sectional survey was conducted among patients attending clinics for STI services. Patients' social-demographic and behavioral information was collected and CT infection was determined by nucleic acid amplification test (NAAT) with self-collected urine specimens. Associated factors were identified using logistic regression.

**Results:**

Among the 8,324 participants, the overall prevalence was 9.0% with 10.7% for males and 8.3% for females respectively. Multivariate analysis showed that aged < 24 [adjusted odds ratio (aOR) = 1.27, 95% confidence interval (CI) = 1.01–1.59], being unmarried (aOR = 1.64, 95%CI = 1.35–2.00), having junior high school or below education level (aOR = 1.47, 95%CI = 1.13–1.91), having no access to health insurance (aOR = 1.27, 95%CI = 1.07–1.51), and being positive for *Neisseria gonorrhoeae* (NG, aOR = 4.49, 95%CI = 3.25–6.21) were significantly associated with CT infection.

**Conclusion:**

We found that CT infection is prevalent among patients seeking clinic-based STI services in Southern China. Targeted interventions could be implemented for patients with a higher risk of CT infection including those aged < 24, being unmarried, having junior high school or below education level, having no access to health insurance, and being positive for NG. In addition, routine CT screening could be considered a public health strategy by the government.

## Introductions

Chlamydia caused by *Chlamydia trachomatis* (CT) is the most common sexually transmitted infection (STI) (1) which can cause significant morbidity, particularly in women. Due to the reason that up to 85% of cases in women and men are asymptomatic (2), CT infection often remains undiagnosed and untreated. Untreated CT infection can lead to pelvic inflammatory disease (PID), chronic pelvic pain, ectopic pregnancy, and tubal factor infertility in women (3), urethritis, epididymitis, and other complications including infertility in men (4). CT infection has become a main global public health concern. Based on the estimation of the World Health Organization (WHO) in 2016, there were an estimated 127.2 million new cases of CT infection per year (5). Data from surveillance programmes in the United States (6), the United Kingdom (UK) (7), and Canada (8) indicated an increasing trend of CT infection in recent years. Based on the national case-reporting system in China, the reported incidence of CT infection has increased from 35.8/100,000 in 2011 to 37.1/100,000 in 2015 (9). However, the burden of CT infection in many parts of China is unknown because of the significant under-reporting of the infections, particularly asymptomatic infections, in health facilities. To provide baseline data for estimating the disease burden, developing intervention programmes, and planning for resource allocations, we carried out a preliminary cross-sectional survey to estimate the prevalence of the infection and explored the factors associated with the infection among patients attending clinics for STI services in Southern China.

## Materials

### Study setting and population

The survey was a cross-sectional study in Shenzhen, a “special economic zone” located in south coastal China and adjacent to Hong Kong. The city has witnessed an alarming increase in its economy, migration of population, and the spread of syphilis and other STIs (10). The survey was conducted using a stratified sampling strategy to recruit potential participants. There are 10 administrative districts in Shenzhen, and 6 of them were randomly selected to do the survey. Four hospitals that reported a high number of STI cases in the previous year were selected in each district and except for one district with only two hospitals. During the period of the survey (from April 15 to May 16, 2018), the first 15 patients attending clinics at departments of dermatology, gynecology, urology, and andrology for seeking STI services were invited to participate in the study according to the eligibility criteria. The criteria included being a patient aged 18–49 years old, seeking STI-related services, and having not any antibiotic use in the last 2 weeks. Written informed consent should be obtained before the survey can be conducted.

### Questionnaire interview and specimen collection

An interview with a structured questionnaire was conducted by the physician to collect demographic and behavioral data as well as clinical findings. After completing the interview, participants were asked to provide a self-administered 15–30 mL first-catch urine specimen. A research nurse was assigned to check the integrity of questionnaire information and instruct participants on specimen collection. Urine specimens were collected using the Cobas1 urine specimen collection kit (Roche P/N 05170486190) according to the manufacturer's instructions. The specimens were temporarily stored at 4°C at the local laboratory for 10 days maximum before being transported to a central laboratory for testing.

### Laboratory assays

At the central laboratory, DNA was extracted and purified from the urine specimens by an automated magnetism nucleic acid isolation method using the MagNA Pure 96 System (Roche, Switzerland) according to the manufacturer's instructions. The extracted DNA was further evaluated for CT and *Neisseria gonorrhoeae* (NG) based on polymerase chain reaction (PCR) of the Cobas® 4800 System (Roche, Switzerland) using Cobas® 4800 CT/NG Amplification/Detection Kit. Diagnosis reagents and supplies were preserved under the requested condition. Laboratory performance was run according to standard operating procedures (SOPs). CT or NG infection was defined as having a positive PCR for CT or NG.

### Statistical analyses

All data from questionnaires and laboratory tests were double-entered into the computer to establish a database using Epidata software (V.3.1, Denmark). The Epidata 3.1 dataset was subsequently transferred to the IBM SPSS Statistics for Windows Version 23.0 (IBM Corp., Armonk, NY) for statistical analyses. Univariate analysis was used to determine the association between variables and CT infection. Crude odds ratio (cOR), adjusted odds ratio (aOR), and 95% confidence interval (CI) were calculated. To adjust for potential confounders, all factors associated with the infection at *P* < 0.2 in univariate analysis were included in multivariable logistic regression analysis using a backward stepwise procedure. Variables significant at *P* < 0.05 were considered the factors independently associated with the infections.

### Patient and public involvement statement

Participation in this study was voluntary and the questionnaire was anonymous. Confidentiality of the study data can protect the privacies of the participants. Participants who tested positive for CT and/or NG were contacted privately by the research team members for further diagnosis, treatment, and other interventions at the STI clinic in Shenzhen Center for Chronic Disease Control. Partner notification was conducted according to the routine process in the clinic.

## Results

### Participant characteristics

Out of the 8,444 patients who provided urine specimens, 120 did not participate in a questionnaire interview. Therefore, a total of 8,324 (98.6%) participants were included in the final analyses. The average age was 32.1 years old (standard deviation [SD] 7.3 years), and 14.3% (1183/8281) of them were younger than 24 years old. About one-third of the participants were males (30.9%, 2567/8309). Most of the participants were married (75.1%, 6207/8269), heterosexuals (98.2%, 8039/8189), migrants (73.7%, 6022/8173), and living in Shenzhen for more than 2 years (77.3%, 6331/8189). 38.8% (3232/8324) of the participants had a monthly income above 5,000 CNY (China Yuan), most of whom were workers (25.4%, 2099/8264) and government employees (25.6%, 2119/8264). Less than one-fifth (17.7%, 1457/8223) finished their education in college or above. 37.6% (3096/8229) had no hold of health insurance and 36.2% (2973/8203) had casual sex in the last 3 months. 21.7% (1787/8231) knew about CT. 2.4% (196/8324) had positive *Neisseria gonorrhoeae* detection. More details are shown in Table 1.

**Table 1:** Socio–demographic, and behavioral data of survey participants at baseline (*n* = 8324).

**Characteristic**	Frequency^*^(%)	**Characteristic**	Frequency^*^(%)
**Sex**		**Occupation**	
Male	2567 (30.9)	Workers	2099 (25.4)
Female	5742 (69.1)	Entertainment services	129 (1.6)
**Age in years**		Businessman/Catering services	1606 (19.4)
≤ 24	1183 (14.3)	Government employees	2119 (25.6)
>24	7098 (85.7)	Household/Unemployed	1267 (15.3)
**Marital status**		Others	1044 (12.6)
Single and others	2062 (24.9)	**Education level**	
Married	6207 (75.1)	Junior high school or below	2636 (32.1)
**Sexual orientation**		Senior high school	2407 (29.3)
Heterosexuality	8039 (98.2)	Junior college	1723 (21.0)
Homo–or bi–sexuality	150 (1.8)	College or above	1457 (17.7)
**Residence**		**Hold of health insurance**	
Shenzhen	2151 (26.3)	Yes	5133 (62.4)
Other places	6022 (73.7)	No	3096 (37.6)
**Duration in Shenzhen**		**Casual sex in the last 3 months**	
<2 years	1858 (22.7)	Yes	2973 (36.2)
≥2 years	6331 (77.3)	No	5230 (63.8)
**Monthly income (CNY)**		**Knowledge about CT**	
<3000	1672 (20.1)	Unknown	6444 (78.3)
3001–5000	2775 (33.3)	Known	1787 (21.7)
5001–10000	2666 (32.0)	***Neisseria gonorrhoeae*** **detection**	
>10000	566 (6.8)	Negative	8128(97.6)
Not quite clear	645 (7.7)	Positive	196(2.4)

* The sum of participants in some items may be less than the total number of 8324 because some participants did not respond to these items. CNY, China Yuan, the minimum wage in Shenzhen, China in 2018 was 2200 CNY. CT, *Chlamydia trachomatis*; %, Constituent ratio.

### Prevalence and associated factors of CT infection

Among the 8,324 participants, 751 people were identified as CT positive, giving an overall prevalence of 9.0% with 10.7% for males and 8.3% for females respectively. The highest prevalence was detected among people aged younger than 24 (15.4%). CT infection of participants (positive and negative) was regarded as the dependent variable and the other factors were used as the independent variables in the logistic regression model. In the univariate analyses, government employees (cOR = 0.75, 95%CI = 0.57–0.97) were significantly associated with a decreased risk of CT infection (*P* < 0.05). Meanwhile, male (cOR = 1.33, 95%CI = 1.14–1.56), aged < 24 (cOR = 2.10, 95%CI = 1.76–2.52), being unmarried (cOR = 2.10, 95%CI = 1.80—-.46), migrants (cOR = 1.40, 95%CI = 1.16–1.68), residing in Shenzhen for < 2 years (cOR = 1.44, 95%CI = 1.22–1.71), entertainment service providers (cOR = 1.73, 95%CI = 1.03–2.91), having junior college or below education level, having no access to health insurance (cOR = 1.59, 95%CI = 1.37–1.85), having casual sex in the last 3 months (cOR = 1.21, 95%CI = 1.04–1.41), having no knowledge about CT (cOR = 1.23, 95%CI = 1.01–1.49) and positive for NG (cOR = 5.52, 95%CI = 4.06–7.49) were significantly associated with an increased risk of CT infection (*P* < 0.05). Sexual orientation and monthly income were not significantly associated with CT infection (*P* > 0.05).

In the univariate analyses, 11 variables were associated with CT infection at *P* < 0.20 (Table 2). In the multivariate analyses using these 11 variables as independent variables and potential interactions between these variables, the following factors were found to be significantly associated with CT infection: aged < 24 (aOR = 1.27, 95%CI = 1.01–1.59), being unmarried (aOR = 1.64, 95%CI = 1.35–2.00), having junior high school or below education level (aOR = 1.47, 95%CI = 1.13–1.91), having no access to health insurance (aOR = 1.27, 95%CI = 1.07–1.51), and being positive for NG (aOR = 4.49, 95%CI = 3.25–6.21). More details are shown in Table 2.

**Table 2:** Prevalence and associated factors of chlamydial infection among patients seeking clinic–based STI services in Shenzhen, China.

**Associated factors**	**Prevalence** **of CT (%)**	**Univariate**		**Multivariate**	
		**cOR (95% CI)**	***P*** **-Value**	**aOR (95% CI)**	***P*** **-Value**
**Sex**					
Male	10.7	1.33(1.14–1.56)	0.000^*^	–	
Female	8.3	1.00		1.00	
**Age in years**					
≤ 24	15.4	2.10(1.76–2.52)	0.000^*^	1.27(1.01–1.59)	0.042^*^
>24	8.0	1.00		1.00	
**Marital status**					
Single or others	14.3	2.10(1.80–2.46)	0.000^*^	1.64(1.35–2.00)	0.000^*^
Married	7.3	1.00		1.00	
**Residence**					
Shenzhen	7.2	1.00		1.00	
Other places	9.8	1.40 (1.16–1.68)	0.000^*^	–	
**Duration in Shenzhen**					
<2 years	11.2	1.44(1.22–1.71)	0.000^*^		
≥2 years	8.3	1.00		1.00	
**Occupation**					
Workers	9.9	1.03(0.80–1.33)	0.801	–	
Entertainment services	15.5	1.73(1.03–2.91)	0.038^*^	–	
Businessman/Catering services	10.3	1.08(0.83–1.40)	0.560	–	
Government employees	7.3	0.75(0.57–0.97)	0.028^*^	–	
Household/Unemployed	7.8	0.80(0.60–1.07)	0.133	–	
Others	9.6	1.00		1.00	
**Education level**					
Junior high school or below	10.1	1.58(1.24–2.01)	0.000^*^	1.47(1.13–1.91)	0.004^*^
Senior high school / technical secondary school	9.5	1.47(1.15–1.88)	0.002^*^	1.26(0.97–1.64)	0.089
Junior college	8.7	1.34(1.03–1.74)	0.032^*^	1.25(0.95–1.66)	0.111
College or above	6.7	1.00		1.00	
**Hold of health insurance**					
Yes	7.5	1.00		1.00	
No	11.5	1.59(1.37–1.85)	0.000^*^	1.27(1.07–1.51)	0.007^*^
**Casual sex in the last 3 months**					
Yes	10.1	1.21(1.04–1.41)	0.015^*^	–	
No	8.5	1.00		1.00	
**Knowledge about CT**					
Unknown	9.4	1.23(1.01–1.49)	0.036^*^	1.23(1.00–1.51)	0.054
Known	7.8	1.00		1.00	
***Neisseria gonorrhoeae*** **detection**					
Negative	8.4	1.00		1.00	
Positive	33.7	5.52(4.06–7.49)	0.000^*^	4.49(3.25–6.21)	0.000^*^

* P < 0.05. cOR, crude odds ratio; aOR, adjusted odds ratio; CI, confidence interval; CT, *Chlamydia trachomatis*.

## Discussions

The findings from our study indicated a high prevalence of CT infection among both male and female patients attending clinics in departments of dermatology, gynecology, urology, and andrology in Shenzhen and highlighted the risk factors associated with the infection in this population. STI surveillance plays an important role in measuring the magnitude of the STI burden in the general and target populations to assist in programme planning, monitoring trends over time and identifying emerging infections and outbreaks, providing data to advocate for mobilization of resources, and assisting in evaluating the effectiveness of the response (11). Prevalence assessment is one of the core components of WHO–recommended STI surveillance programming.

Our study was a cross–sectional study focusing on genital CT infection with the largest sample size of patients seeking clinic–based STI services in China. Among 8,324 patients, the total prevalence of CT infections was 9.0% with 10.7% for males and 8.3% for females respectively, which was higher than the general population of 2.1% for males and 2.6% for females in China (1999–2000) (12). This CT prevalence was also higher than that reported in many high–income countries (13), such as 1.7% in the US (14), 1.5% in the UK (15), and 1.7% in France (16) among the general population used by Urine NAAT. Compare with high–risk populations, such as 8.5% among cross–border truck drivers in Hong Kong (17), and 6.5% among MSM in Jiangsu Province (18), this CT prevalence remained at a high level. The relatively high prevalence of CT infection further emphasized the importance of urgently implementing comprehensive interventions among patients seeking clinic–based STI services.

The findings in our study showed the highest prevalence (15.4%) was detected among patients seeking clinic–based STI services aged younger than 24. After controlling for confounding factors, aged younger than 24 was still significantly associated with an increased risk of CT infection. This finding was similar to the findings of the US CDC, which was reported in 2013. The highest incidence of CT in the US was among people aged between 14 and 24 years (19). This result may indicate that patients of this age group had more exposure to new infections due to greater sexual activity and lower education on STI prevention. Opportunistic screening for CT among young sexually active adults had been recommended in many high–income countries including the USA, the UK, Australia, Sweden, Denmark, and Norway (20–22). The UK had run a nationwide program called the National Chlamydia Screening Programme (NCSP) (23), which targeted all sexually active men and women under 25 years of age for annual chlamydia screening through various clinical and non–clinical settings. It would be a feasible measure to develop a CT screening strategy for young people in China, which could be started in economically developed regions such as Shenzhen city.

In addition, participants that were single/divorced/widowed were almost 50% more likely to have a CT infection than those who were married. This was consistent with the results of Wong WC et al. (24) and Walsh MS et al. (25) studies. It could be seen that maintaining loyal marital status was an effective protective factor for CT infection after excluding age, not having casual sex in the last 3 months, and other confounding factors. Moreover, our results showed that a positive for NG was significantly associated with an increased risk of CT infection. This was consistent with the findings of Guangdong Province (26). Therefore, it would be better to suggest patients have a CT infection test when they need to have an NG detection.

Regarding the educational level, we found that participants with lower educational levels were at higher risk of incident CT infection, and this association remained after adjusting for age and occupation. Similar results were obtained in other studies (27–29). In addition, we found a higher prevalence in patients who had no hold of health insurance. Poor healthcare–seeking behavior associated with higher infection rates, lower partner referral, or inadequate care had been reported for people with lower socioeconomic status in many countries (30–32). A German study showed that the higher prevalence in groups with low or medium social status could have a lower chance of testing CT and lower healthcare use because a quarterly fee had to be paid by persons, which will pose a possible barrier for people with lower income (33–35). It might be useful to reduce the CT infection of this population by strengthening the publicity and education of CT among the low–education population and reducing the cost of CT screening and treatment.

However, our study had some limitations. First, the study did not investigate the number of sexual partners and condom use of STD patients in the last month. As a result, the CT infection situation cannot be analyzed in depth of the patient's sexual behaviors. Second, due to the lower number of CT–reported cases in private hospitals, it cannot be compared with public hospitals and other private hospitals, which have a higher number of CT–reported cases. Third, Shenzhen has a relatively open–minded sexual attitude, and it is also an economically developed area with a relatively higher young population. Thus, the result of the study cannot represent the health situation of chlamydial infection in the whole country, and cannot be compared with other cities with a lower socioeconomic status.

In conclusion, CT infection is prevalent among patients seeking clinic–based STI services, particularly those aged younger than 24 in Shenzhen, China. Furthermore, patients that were single/divorced/widowed, positive for NG, with low educational level, and having no hold of health insurance were significantly associated with an increased risk of CT infection. It is strongly recommended that clinicians perform key screening and interventions for CT in these populations. At the same time, these findings suggest that CT–integrated prevention and control projects could be considered in routine public health services by the government. However, the high prevalence of CT is not enough for the screening strategy. Efforts are needed in future studies on several areas such as the cost–effectiveness of screening strategies, the burden of disease estimates, the capacity of CT screening in hospitals, the willingness of being screened of patients, and the knowledge of CT in patients.

## Data availability statement

The original contributions presented in the study are included in the article/supplementary material, further inquiries can be directed to the corresponding author.

## Author contributions

HW, YC, and XC conceived and designed the study. FH, CZ, LW, JY, and RW supervised the data and samples collection. HW, YC, XC, CZ, LW, JY, JL, YL, and NN performed the research. HW, RW, YL, YC, and XC analyzed, interpreted the results, and were the major contributors in writing the manuscript. All authors read and approved the final manuscript.

## Funding

This work was supported by the Sanming project of Medicine in Shenzhen (grant number SZSM201611077).

## Conflict of interest

The authors declare that the research was conducted in the absence of any commercial or financial relationships that could be construed as a potential conflict of interest. The reviewer GF declared a shared affiliation with one of the author XC to the handling editor at time of review.

## Publisher's note

All claims expressed in this article are solely those of the authors and do not necessarily represent those of their affiliated organizations, or those of the publisher, the editors and the reviewers. Any product that may be evaluated in this article, or claim that may be made by its manufacturer, is not guaranteed or endorsed by the publisher.

## References

[1] World Health Organization. Global incidence and prevalence of selected curable sexually transmitted infections: 2008. Reproduct Health Matters. (2012) 20:207–9. 10.1016/S0968-8080(12)40660-7

[2] Peipert JF. Clinical practice. Genital chlamydial infections. N Engl J Med. (2003) 349:2424–30. 10.1056/NEJMcp030542 14681509

[3] Haggerty CL Ness RB. Epidemiology, pathogenesis and treatment of pelvic inflammatory disease. Expert Rev Anti Infect Ther. (2006) 2:235–47. 10.1586/14787210.4.2.235 16597205

[4] Wang QQ Liu QZ Xu JH. Treatment and Prevention of Sexually Transmitted Disease. Shanghai: Shanghai Scientific & Technical Publishers. (2014) p. 104–6.

[5] Rowley J Hoorn S V Korenromp E Low N Taylor MM. Chlamydia, gonorrhoea, trichomoniasis and syphilis: Global prevalence and incidence estimates, 2016[J]. Bull World Health Organ. (2019) 97:548–62. 10.2471/BLT.18.228486 31384073 PMC6653813

[6] Centers For Disease Control and Prevention. Sexually Transmitted Disease Surveillance 2014. Atlanta: U.S. Department of Health and Human Services. (2015).

[7] European Centre for Disease Prevention and Control. Sexually Transmitted Infections Surveillance in Europe Annual Report No. 2. (2012).

[8] Public Health Agency of Canada. *Report on Sexually Transmitted Infection in Canada*. (2012). Available online at: http://www.phac-aspc.gc.ca/sti-its-surv-epi/rep-rap-2012/rep-rap-1-eng.php/2 (accessed May 13, 2019).

[9] Chen XS. The Epidemic of Urogenital Infection with Chlamydia Trachomatis in China and the World. Chin Med Abs Dermatol. (2016) 03:265–69.

[10] Dai W Luo Z Xu R Zhao G Tu D Yang L. Prevalence of HIV and syphilis co-infection and associated factors among non-commercial men who have sex with men attending a sexually transmitted disease clinic in Shenzhen, China. BMC Infect Dis. (2017) 17:2187. 10.1186/s12879-017-2187-1 28100187 PMC5241916

[11] World Health Organization. Standard protocol to assess prevalence of gonorrhoea and chlamydia among pregnant women in antenatal care clinics. (2018). Available online at: https://www.who.int/publications/i/item/9789241514675/ (accessed June 15, 2019).

[12] Parish WL Laumann EO Cohen MS Suiming P Heyi Z Irving H. Population-based study of chlamydial infection in China: a hidden epidemic. JAMA.. (2003) 289:1265–73. 10.1001/jama.289.10.1265 12633188

[13] Shelagh M R Karin A Sarah C W Ingrid V F VDB Jan VB Helen W. Genital chlamydia prevalence in Europe and non-European high income countries: systematic review and meta-analysis. PLoS ONE. (2015) 10:e0115753. 10.1371/journal.pone.0115753 25615574 PMC4304822

[14] Torrone E Papp J Weinstock H. Prevalence of Chlamydia trachomatis genital infection among persons aged 14-39 years–United States, 2007-2012. Mmwr Morb Mortal Wkly Rep. (2014) 63:834–8. 10.1186/1471-2458-14-1004 25254560 PMC4584673

[15] Sonnenberg P Clifton S Beddows S Field N Soldan K Tanton C. Prevalence, risk factors, and uptake of interventions for sexually transmitted infections in Britain: findings from the National Surveys of Sexual Attitudes and Lifestyles (Natsal). Lancet. (2013) 382:1795–806. 24286785 10.1016/S0140-6736(13)61947-9 PMC3899025

[16] Goulet V Barbeyrac BD Raherison S Prudhomme M Semaille C Warszawski J. Prevalence of Chlamydia trachomatis: results from the first national population-based survey in France. Sex Transm Infect. (2010) 86:263–70. 10.1136/sti.2009.038752 20660590

[17] Leung PHM Boost MV Lau JTF Wong ATY Pang M Ng TK. Prevalence and risk factors for Chlamydia trachomatis infection among cross-border truck drivers in Hong Kong. Sex Transm Infect. (2009) 85:27–9. 10.1136/sti.2008.031880 18708483

[18] Geng-Feng F Ning J Hai-Yang H Tanmay M Yue-Ping Y Sanchita M. The epidemic of HIV, syphilis, chlamydia and gonorrhea and the correlates of sexual transmitted infections among men who have sex with men in Jiangsu, China, 2009. PLoS ONE. (2015) 10:e0118863. 10.1371/journal.pone.0118863 25775451 PMC4361608

[19] Satterwhite CL Torrone E Meites E Dunne EF Mahajan R Ocfemia MCB. Sexually transmitted infections among US women and men. Sex Transm Dis. (2013) 40:187–93. 10.1097/OLQ.0b013e318286bb53 23403598

[20] Lanjouw E Ouburg S de Vries HJ Stary A Radcliffe K Unemo M. 2015 European guideline on the management of Chlamydia trachomatis infections. Int J STD AIDS. (2015) 37:721–9. 10.1177/0956462415618837 26608577

[21] Workowski KA. Centers for disease control and prevention sexually transmitted diseases treatment guidelines. Clin Infect Dis. (2015) 61 Suppl 8:S759–62. 10.1093/cid/civ771 26602614

[22] Low N Cassell JA Spencer B Bender N Martin Hilber A Van Bergen J. Chlamydia control activities in Europe: cross-sectional survey. Eur J Public Health. (2012) 22:556–61. 10.1093/eurpub/ckr046 21531771

[23] Chandra NL Soldan K Dangerfield C Sile B Duffell S Talebi A. Filling in the gaps: estimating numbers of chlamydia tests and diagnoses by age group and sex before and during the implementation of the English National Screening Programme, 2000 to 2012. Euro Surveill. (2017) 22:30453. 10.2807/1560-7917.ES.2017.22.5.30453 28183393 PMC5388116

[24] Wong WC Zhao Y Wong NS Parish WL Miu HY Yang LG. Prevalence and risk factors of chlamydia infection in Hong Kong: a population-based geospatial household survey and testing. PLoS ONE. (2017) 12:e0172561. 10.1371/journal.pone.0172561 28225805 PMC5321413

[25] Walsh MS Hope E Isaia L Righarts A Niupulusu T Temese SV. Prevalence of Chlamydia trachomatis infection in Samoan women aged 18 to 29 and assessment of possible risk factors: a community-based study. Trans R Soc Trop Med Hyg. (2015) 109:245–51. 10.1093/trstmh/trv014 25732755

[26] Shen HC Huang SJ Qin XL Zhao PZ Lan YY Zou HC. Genital Chlamydia trachomatis infection and associated risk factors in male clients attending sexually transmitted disease clinics in 9 cities in Guangdong province. Zhonghua liu xing bing xue za zhi. (2017) 38:364. 10.3760/cma.j.issn.0254-6450.2017.03.018 28329941

[27] Walker J Tabrizi SN Fairley CK Chen MY Bradshaw CS Twin J. Chlamydia trachomatis incidence and re-infection among young women–behavioural and microbiological characteristics. PLoS ONE. (2012) 7:e37778. 10.1371/journal.pone.0037778 22662220 PMC3360595

[28] Skjeldestad FE Marsico MA Sings HL Nordbo SA Storvold G. Incidence and risk factors for genital Chlamydia trachomatis infection: a 4-year prospective cohort study. Sex Transm Dis. (2009) 36:273–9. 10.1097/olq.0b013e3181924386 19265733

[29] Deogan C Cnattingius S Mansdotter A. Risk of self-reported Chlamydia trachomatis infection by social and lifestyle factors: a study based on survey data from young adults in Stockholm, Sweden. Eur J Contracept Reprod Health Care. (2012) 17:458–67. 10.3109/13625187.2012.729624 23113560

[30] Andrade MV Noronha K Singh A Rodrigues CG Padmadas SS. Antenatal care use in Brazil and India: Scale, outreach and socioeconomic inequality. Health Place. (2012) 18:942–50. 10.1016/j.healthplace.2012.06.014 22832334

[31] Watanabe R Hashimoto H. Horizontal inequity in healthcare access under the universal coverage inJapan 1986–2007. Soc Sci Med. (2012) 75:1372–8. 10.1016/j.socscimed.2012.06.006 22809794

[32] Zhang Q Lauderdale D Mou S Parish WI Laumann EO Schneider J. Socioeconomic disparity in healthcare-seeking behavior among Chinese Women with genitourinary symptoms. J Womens Health. (2009) 18:1833. 10.1089/jwh.2009.1394 19951219 PMC2828239

[33] Simoes E Kunz S Schmahl F. Utilisation gradients in prenatal care prompt further development of the prevention concept. Das Gesundheitswesen. (2009) 71:385. 10.1055/s-0029-1214401 19492278

[34] Mcgarrity LA Huebner DM. Behavioral intentions to HIV test and subsequent testing: the moderating role of sociodemographic characteristics. Health Psychol. (2014) 33:396. 10.1037/a0033072 23795706

[35] Lostao L Regidor E Geyer S Aïach P. Patient cost sharing and social inequalities in access to health care in three western European countries. Soc Sci Med. (2007) 65:367–76. 10.1016/j.socscimed.2007.05.001 17544192

